# Contact Tracing for Influenza A(H1N1)pdm09 Virus–infected Passenger on International Flight

**DOI:** 10.3201/eid2001.120101

**Published:** 2014-01

**Authors:** Ananda G. Shankar, Kulsum Janmohamed, Babatunde Olowokure, Gillian E. Smith, Angela H. Hogan, Valerie De Souza, Anders Wallensten, Isabel Oliver, Oliver Blatchford, Paul Cleary, Sue Ibbotson

**Affiliations:** Health Protection Agency, West Midlands Region, Birmingham, UK (A.G. Shankar, K. Janmohamed, B. Olowokure, G.E. Smith, V. De Souza, S. Ibbotson);; Health Protection Agency, Southwest Region, Gloucester, UK (A.H. Hogan, A. Wallensten, I. Oliver);; Swedish Institute for Communicable Disease Control, Solna, Sweden (A. Wallensten);; NHS National Services Scotland, Glasgow Scotland, UK (O. Blatchford);; Health Protection Agency, Northwest Region, Liverpool, UK (P. Cleary)

**Keywords:** pandemic, influenza A(H1N1)pdm09, in-flight exposure, international flights, contact tracing, transmission, infectious disease, influenza, viruses, aircraft passengers, swine flu, United Kingdom, Mexico

## Abstract

In April 2009, influenza A(H1N1)pdm09 virus infection was confirmed in a person who had been symptomatic while traveling on a commercial flight from Mexico to the United Kingdom. Retrospective public health investigation and contact tracing led to the identification of 8 additional confirmed cases among passengers and community contacts of passengers.

On April 27, 2009, influenza A(H1N1)pdm09 virus infection was confirmed in a passenger who had traveled on a commercial flight from Mexico to the United Kingdom (UK) (*1*). This was the first identified imported case of A(H1N1)pdm09 infection in the UK. The person departed Mexico on April 20, 2009, and arrived in Birmingham, UK, 9.5 hours later on April 21, 2009. We describe the contact-tracing investigation of passengers on the flight and estimate the risk for transmission of A(H1N1)pdm09 virus to the passengers.

## The Study

During the flight aboard a Boeing 767-300 airplane from Mexico to the UK, the index A(H1N1)pdm09 patient (case-patient 1) was seated in the rear cabin. Bulkheads and toilets divided the airplane into 3 sections (front, middle, and rear). On the implicated flight, 282 passenger seats were available. Case-patient 1 is believed to have become symptomatic on April 18, 2009 (2 days before departing Mexico) and continued to be symptomatic during the flight. Reported symptoms were fever, cough, headache, myalgia, and chills. A nasopharyngeal swab sample obtained on April 24 was PCR positive for A(H1N1)pdm09 virus. A traveling companion of case-patient 1 (case-patient 2) was asymptomatic during the flight but symptomatic on April 23, 2009; a nasopharyngeal swab sample obtained on April 25th was PCR positive for A(H1N1)pdm09 virus.

By using information from the airline’s passenger manifest, we identified close contacts of case-patient 1. Close contacts were defined as passengers seated in the same row as or in the 2 rows in front of or behind the row in which case-patient 1 sat. This definition is consistent with World Health Organization guidance for post-flight influenza contact tracing (*2*).

Beginning April 29, 2009, close contacts of case-patient 1 were interviewed by telephone; a structured questionnaire was used. Because case-patients 1 and 2 had the first identified cases of A(H1N1)pdm09 infection in the UK, they had already been extensively interviewed. Thus, we extracted relevant information from those interviews and did not re-interview the patients. Data for these 2 persons are not included in the calculation of post-flight attack rates.

Passengers on the flight were considered to be post-flight case-patients if they had influenza-like illness <7 days after arrival in the UK and had positive test results for A(H1N1)pdm09 virus. Influenza-like illness was defined as fever, measured or subjective, plus >2 of the following signs or symptoms: cough, sore throat, rhinorrhea, myalgia, headache, vomiting, and diarrhea. Using case-patient 1 as the initiator of the chain of transmission, we categorized post-flight case-patients as first-generation case-patients. Persons with cases arising from first-generation cases were categorized as second-generation case-patients. Passengers identified as close contacts but who did not meet these criteria were not regarded as case-patients. To identify other A(H1N1)pdm09 cases in the UK associated with this flight, we reviewed the Health Protection Agency’s First Few Hundred national database (*3*).

Thirty-nine passengers on the flight (all of whom lived in the UK) were identified as close contacts of case-patient 1, and 37/39 were asymptomatic during the flight. The 2 passengers who were symptomatic (cough and subjective fever) during the flight sat within 1 row of case-patient 1; both had test results negative for A(H1N1)pdm09 infection. All close contacts were interviewed within 3 weeks (median 13 days, range 8–20 days) of disembarkation.

Two of the 37 case-patients who were asymptomatic during the flight later tested positive for A(H1N1)pdm09 infection. One of these persons (case-patient 2, the traveling companion of case-patient 1) was seated next to case-patient 1; the other person (case-patient 3) was seated 2 rows behind case-patient 1. Therefore, after excluding case-patient 2, the attack rate for persons identified as first-generation cases and close contacts of case-patient 1 was 1/38 (2.6%, 95% CI 0.5%–13.5%).

Details of 6 additional confirmed cases that were identified after a review of a national database are shown in the Table (cases 4–9). Four of these cases were first-generation cases (cases 4–7). The others (cases 8–9) were classified as second-generation cases and occurred in persons who had not been on the flight and had no travel history but were known to have had direct contact with persons who had been on the flight.

**Table:** Selected characteristics of persons with confirmed cases of influenza A(H1N1)pdm09 virus infection, United Kingdom, April 2009*

Case-patient no.	Symptomatic during flight	No. rows from index case-patient	Day of symptom onset, April 2009, no. days before/after flight
1†	Yes		18th, 3 before
2‡	No	Same	23rd, 2 after
3	No	2 behind	24th, 3 after
4	No	4 in front	26th, 5 after
5	No	4 in front	24th, 3 after
6	No	5 behind	22nd, 1 after
7	No	8 behind	24th, 3 after
8§	NA	NA	25th, 4 after
9¶	NA	NA	26th, 5 after

*Characteristics were determined during flight-related contact tracing. NA, not applicable. †Case-patient 1, the index patient, had the first laboratory-confirmed case of A(H1N1)pdm09 infection in the United Kingdom. ‡Case-patient 2 was the traveling companion of case-patient 1. §Case-patient 8 was not on the flight and is a secondary case-patient who is believed to have been exposed to the virus by case-patient 2. ¶Case-patient 9 was not on the flight and is believed to have been exposed to the virus by a passenger who was on the flight but who had test results negative for A(H1N1)pdm09 virus.

Case-patients 4 and 5 had been seated next to each other in the middle section of the cabin, 4 rows in front of case-patient 1. They were situated directly in front of the bulkhead separating the middle and rear sections of the cabin. Case-patients 6 and 7 were seated within 3 rows of each other and 5 and 8 rows, respectively, behind case-patient 1 in the rear section of the cabin. The attack rate for passengers sitting elsewhere in the plane and not regarded as close contacts of case-patient 1 was 4/238 (1.7%, 95% CI 0.5%–4.3%), whereas the attack rate for all passengers was 5/276 (1.8%, 95% CI 0.8%– 4.2%). Altogether, 4 of the confirmed cases were identified among the 96 passengers seated in the rear section of the cabin, where the attack rate was 4.2% (95% CI 1.2%–10.3%).

## Conclusions

The investigation of passengers on this flight and their contacts identified 9 cases of PCR-confirmed A(H1N1)pdm09 infection: the index case-patient, who had been symptomatic while traveling; 6 other passengers on the same flight; and 2 members of the public who had exposure to persons who had been asymptomatic passengers on the flight. Of the 6 confirmed case-patients on the flight, only 2 (including case-patient 2, the traveling companion of case-patient 1) had been seated within 2 rows of case-patient 1.

It cannot be definitively stated that A(H1N1)pdm09 virus was transmitted from case-patient 1 to other passengers during this flight; however, several reasons support our assumption that such transmission did occur. At the time of disembarkation, there were no known cases of A(H1N1)pdm09 virus infection in the UK, and no other plausible sources of infection were identified. These facts increase the likelihood that the 2 second-generation cases identified are directly attributable to passengers on the flight who were identified as first-generation case-patients. The distribution of possible first-generation cases within the aircraft reflects previous reports describing the in-flight transmission of influenza viruses and A(H1N1)pdm09 virus (*4**–**7*). Symptom onset for the 6 first-generation cases occurred 1–5 days after disembarkation, and although it is possible that infection could have occurred in Mexico at any time before embarkation, this timeline is also within the range for in-flight transmission and consistent with the known epidemiology of A(H1N1)pdm09 virus (*8*).

It is also plausible that this flight was multiply seeded with asymptomatic infected persons. In particular, it is possible that the first-generation case-patients seated >2 rows away from case-patient 1 may have been exposed to A(H1N1)pdm09 virus by other unidentified, infected persons.

This study had limitations. First, contact tracing was limited to close contacts of case-patient 1. Second, not all rear-cabin passengers were tested or interviewed. Last, confirmation was based on PCR testing of a single nasopharyngeal swab sample, so it is possible that some infections were missed, leading to an underestimate of transmission risk.

The results of this study suggest that where in-flight transmission of a novel virus is suspected, restricting contact tracing to passengers within a 2-row zone may result in a failure to identify other cases (*5*,*6*,*9*). Such an outcome may have implications with regard to the global spread of a new disease.
